# Membrane Dynamics and Cation Handling in Ferroptosis

**DOI:** 10.1152/physiol.00029.2023

**Published:** 2024-01-09

**Authors:** Yusuke Hirata, Eikan Mishima

**Affiliations:** ^*1*^Laboratory of Health Chemistry, Graduate School of Pharmaceutical Sciences, Tohoku University, Sendai, Japan; ^*2*^Institute of Metabolism and Cell Death, Helmholtz Zentrum München, Neuherberg, Germany; ^*3*^Division of Nephrology, Rheumatology and Endocrinology, Tohoku University Graduate School of Medicine, Sendai, Japan

**Keywords:** cell death, ion channels, lipid peroxidation, oxidative stress, Piezo1

## Abstract

Ferroptosis, a regulated cell death hallmarked by excessive lipid peroxidation, is implicated in various (patho)physiological contexts. During ferroptosis, lipid peroxidation leads to a diverse change in membrane properties and the dysregulation of ion homeostasis via the cation channels, ultimately resulting in plasma membrane rupture. This review illuminates cellular membrane dynamics and cation handling in ferroptosis regulation.

## Introduction: Membrane Rupture in Regulated Cell Death

Cell death constitutes an essential biological process crucial for various aspects of life, including tissue homeostasis and disease progression. Diverging markedly from accidental cell death, a biologically uncontrolled process of cells caused by lethal physical, chemical, or mechanical stress, regulated cell death is executed through meticulously orchestrated molecular machinery and highly organized signaling cascades. These mechanisms play a pivotal role in maintaining organismal homeostasis under both physiological and pathological conditions. Recent research advances have shed light on the distinct machinery and (patho)physiological roles of different forms of regulated cell death such as apoptosis, necroptosis, pyroptosis, and ferroptosis (1).

The plasma membrane serves as a primary barrier guarding cells against the extracellular environment, making it central to homeostatic maintenance. The rupture of the plasma membrane unequivocally leads to cell death, marking the cataclysmic end point for cellular life. Notably, this membrane rupture is the hallmark of necrotic cell death, except in apoptosis, where plasma membrane integrity is preserved (2). Despite the historical view of necrotic cell death as uncontrolled and passive, it is now evident that intrinsic programs can drive regulated necrosis, encompassing necroptosis and ferroptosis. Additionally, plasma membrane rupture in apoptotic cells undergoing secondary necrosis is also intrinsically regulated (3).

In the process of regulated necrosis, the translocation of channel and/or pore proteins to the plasma membrane initiates changes in membrane permeability, culminating in the loss of plasma membrane integrity and rupture (4). However, it is noteworthy that the signals responsible for membrane dynamics during the cell death process vary among different cell death modalities, distinguishing one from another. Thus, it is important to comprehend the molecular processes that occur on or within the plasma membrane in each form of cell death. Nonetheless, despite the comprehensive examination of necroptosis and pyroptosis, which represent major forms of regulated cell death, our understanding of membrane processes in ferroptosis remains elusive. This review is dedicated to shedding light on the dynamics of the cellular membrane, with a specific focus on ferroptosis.

## Ferroptosis

Ferroptosis is a form of nonapoptotic regulated cell death hallmarked by excessive (phospho)lipid peroxidation mediated by iron (5). Mechanistically, it has been considered excessive (phospho)lipid peroxidation in cellular membranes caused by disruption of the antioxidant defense system, disrupting membrane integrity and ultimately leading to cell death with plasma membrane rupture. Although the term “ferroptosis” was introduced in 2012 (6), cell death displaying ferroptosis-like features had been observed long before the terminology was coined. Previously documented instances of oxidative stress-induced cell death resulting from factors such as glutathione (GSH) deprivation, excessive glutamate exposure, or genetic loss of glutathione peroxidase 4 (GPX4) are now recognized as ferroptosis (7–9). Over the past decade, research has identified cellular processes that either protect against or promote lipid peroxidation and ferroptosis. Cells employ a range of enzymatic and nonenzymatic systems to monitor and safeguard against deleterious lipid peroxidation of cellular membranes (10). In addition, recent findings have implicated ferroptosis in various pathological settings, including acute organ injury, neurodegenerative diseases, and its role in promoting tumor suppression (5). Thus, pharmacological intervention targeting ferroptosis, whether inducing or inhibiting it, holds attractive promise for treating diseases associated with ferroptosis, offering therapeutic approaches for certain cancer states characterized by heightened susceptibility to ferroptosis (11, 12).

## Regulatory Pathway of Ferroptosis

The hallmark of ferroptosis is the excessive and uncontrolled occurrence of cellular phospholipid hydroperoxide (PLOOH), which is mediated by the presence of iron and can be induced by disruption of the antioxidant defense systems. Thus, sensitivity of the cell to ferroptosis is considered to be determined by three main factors: *1*) the lipid peroxidation surveillance system, *2*) the extent of lipid contents in the cellular membrane, and *3*) the amount of labile iron pool.

**FIGURE 1.**
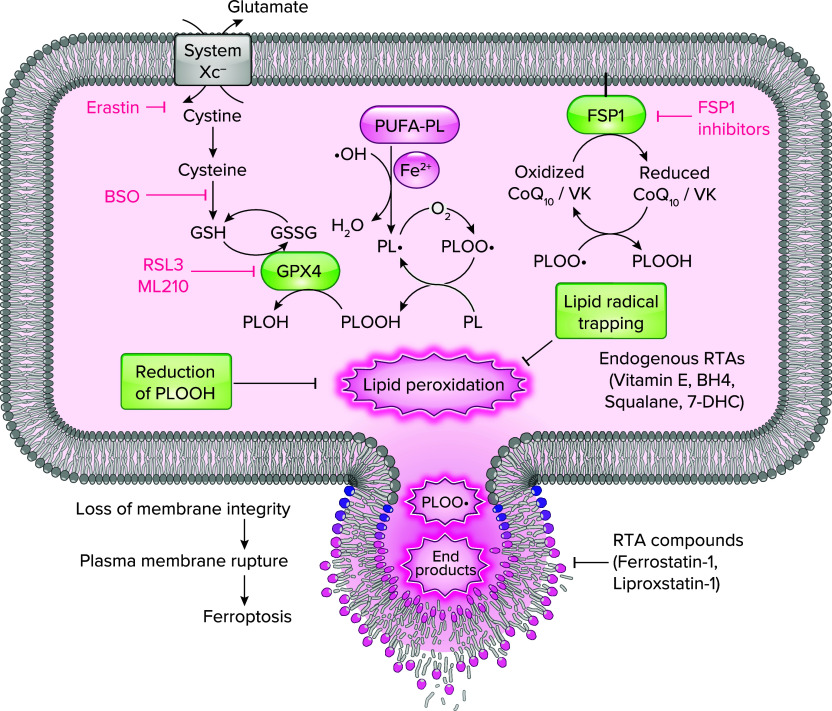
Regulatory pathways of ferroptosis Ferroptosis, characterized by extensive lipid peroxidation, is considered to be initiated by Fenton reaction involving hydroxyl radical (•OH), which has the ability to remove a bis-allylic hydrogen atom from a polyunsaturated fatty acid (PUFA) incorporated within phospholipids (PLs), the primary constituents of lipid bilayers. This results in generation of a phospholipid radical (PL•). Upon subsequent interaction with molecular oxygen (O_2_), a phospholipid peroxyl radical (PLOO•) is generated, which in turn extracts a hydrogen atom from another PUFA-PL, leading to the formation of phospholipid hydroperoxide (PLOOH). Extensive peroxidation of PL and the generation of lipid radicals, including PLOO•, disrupt membrane integrity, eventually triggering ferroptosis by plasma membrane rupture. *Left*: glutathione (GSH) is synthesized from cysteine, which is sourced from cystine via the cystine/glutamate antiporter, system Xc^−^. Glutathione peroxidase 4 (GPX4) utilizes GSH to efficiently reduce toxic PLOOHs, converting them into corresponding alcohol (PLOH). Through detoxification of toxic PLOOHs, GPX4 effectively prevents ferroptosis. Oxidized GSH (GSSG) is regenerated by glutathione reductase to yield GSH. *Right*: in the ferroptosis suppressor protein 1 (FSP1) pathway, FSP1 reduces coenzyme Q10 (CoQ10) and vitamin K (VK) to their reduced forms. The reduced forms of CoQ10 and vitamin K in turn suppress phospholipid peroxidation of lipid bilayers by trapping lipid radicals. Endogenous radical trapping antioxidants (RTAs), including vitamin E, tetrahydrobiopterin (BH_4_) and 7-dehydrocholesterol (7-DHC), and RTA compounds, including ferrostatin-1 and liproxistatin-1, also have the ability to prevent ferroptosis by trapping lipid radicals. Erastin, l-buthionine sulfoximine (BSO), and GPX4 inhibitors (RSL3 and GPX4) induce ferroptosis by inhibiting Xc^−^, GSH synthesis, and GPX4, respectively. FSP1 inhibitors sensitize cells to ferroptosis.

Within the lipid peroxidation surveillance system, cyst(e)ine/glutathione (GSH)/GPX4 pathway is the prime defense system suppressing ferroptosis (FIGURE 1) (13, 14). GPX4, which is the master regulator of ferroptosis, detoxifies potentially toxic oxidized phospholipids (i.e., PLOOH) into corresponding alcohol by enzymatic reduction consuming GSH as a cofactor, and thereby effectively averting lipid peroxidation and rupturing of the plasma membrane for prevention of ferroptosis (15). Since a sufficient amount of GSH is essential for the optimal functioning of GPX4, cysteine and its oxidized dimeric form cystine, which are the substrates limiting GSH synthesis, have key roles in cellular antioxidant defenses. Most cellular cystine is imported through cystine/glutamate antiporter (system Xc^−^), which exchanges extracellular cystine for intracellular glutamate (16). Imported cystine is converted to its reduced form cysteine in the cells, which is then used for GSH biosynthesis. Thus, cyst(e)ine, GSH, and GPX4 form the essential pathway for preventing ferroptosis.

More recently, other GSH-independent ferroptosis surveillance pathways have been identified. Ferroptosis suppressor protein 1 (FSP1; encoded by *AIFM2* gene), along with extramitochondrial ubiquinone (CoQ10) or vitamin K, protects against lipid peroxidation by neutralizing lipid radicals (17–19). Mechanistically, the reduced forms of CoQ10 and vitamin K mediated by FSP1 function as lipid radical scavengers, thereby preventing ferroptosis (17, 20). Radical-trapping antioxidants endogenously synthesized, such as vitamin E, tetrahydrobiopterin, squalene, and 7-dehydrocholesterol, also suppress lipid peroxidation and function to prevent ferroptosis (21–23).

The unrestrained generation of polyunsaturated fatty acid (PUFA)-containing PLOOH in cellular membranes is the ultimate trigger of lipid peroxidation involved in ferroptosis (24). Unlike saturated fatty acids and monounsaturated fatty acids, PUFA chains in membrane lipids are by far more susceptible to lipid oxidation. Thus, contents of PUFAs esterified in phospholipid membrane bilayers are a crucial factor for ferroptosis regulation. As such, enzymes involved in PUFA and phospholipid metabolism [such as acyl-CoA synthetase long chain family member (ACSL)4 and ACSL1] (25, 26) as well as plasma membrane repair [such as Ca^2+^-independent (i)phospholipase A_2_ (PLA_2_)β and lipoprotein-associated (Lp-)PLA_2_] (27, 28) modulate the cells’ vulnerability to ferroptosis by modulating the amount of (oxidized)-PUFA.

As the name ferroptosis implies, iron is an essential element in ferroptosis (29). Intracellular redox-active iron facilitates ferroptosis by catalyzing the formation of lipid radicals, although the precise role of iron has been debated. Mechanistically, PLOOHs react with ferrous iron (Fe^2+^) to generate lipid radicals, driving the lipid peroxidation chain reaction (30). When the Fenton reaction involving Fe^2+^ occurs, it leads to the generation of highly reactive oxygen radicals, specifically HO•, which have the capability to extract a bis-allylic hydrogen atom from PUFA, resulting in the formation of a carbon-centered radical. Subsequently, upon interaction with molecular oxygen, the produced peroxyl radical (HOO•) can readily abstract another hydrogen atom from an adjacent acyl chain. If this lipid peroxidation chain reaction persists without interruption by the aforementioned cellular defense mechanisms, it ultimately triggers ferroptosis. Consequently, a variety of cellular processes that regulate the import, export, storage, and release of cellular labile iron alter cell sensitivity to lipid peroxidation and ferroptosis (29).

## Inducers and Inhibitors of Ferroptosis

There is a growing interest in the development of new chemical entities that act as ferroptosis suppressors or inducers. In principle, ferroptosis can be suppressed typically by scavenging lipid radicals with radical-trapping antioxidants, depleting iron, or modulating pathways that regulate ferroptosis (FIGURE 1). Considering that ferroptosis is driven by phospholipid peroxidation, administration of lipophilic radical-trapping antioxidants, such as ferrostatin-1, liproxstatin-1, and α-tocopherol, is a key strategy for preventing ferroptosis (30). A second strategy for blocking lipid peroxidation is to deplete the labile iron pool with iron chelators such as deferoxamine. A strategy involves targeting enzymes responsible for ferroptosis regulation such as the upregulation of GPX4 and FSP1 and the downregulation of the ferroptosis-promoting genes, such as ACSL4.

In contrast, for induction of ferroptosis, pharmacological and genetic interventions disrupting cyst(e)ine/GSH/GPX4 pathway are utilized (6). For example, erastin is a strong inhibitor of system Xc^−^. l-buthionine sulfoximine (BSO) causes GSH depletion (31). *1S,3R*-RSL3 (RSL3) and ML210 are GPX4 inhibitors (14) (FIGURE 1). These compounds induce ferroptosis by disrupting cyst(e)ine/GSH/GPX4 pathway. Genetic depletion of *GPX4* also induces ferroptosis, and it is also used for in vivo ferroptosis models (13, 19, 32). Pharmacological inhibition of FSP1 has also been shown to sensitize cells to ferroptosis in synergy with inhibitors of GPX4 or system Xc^−^ (33, 34).

## Effects of Lipid Peroxidation on Membrane Properties

**FIGURE 2.**
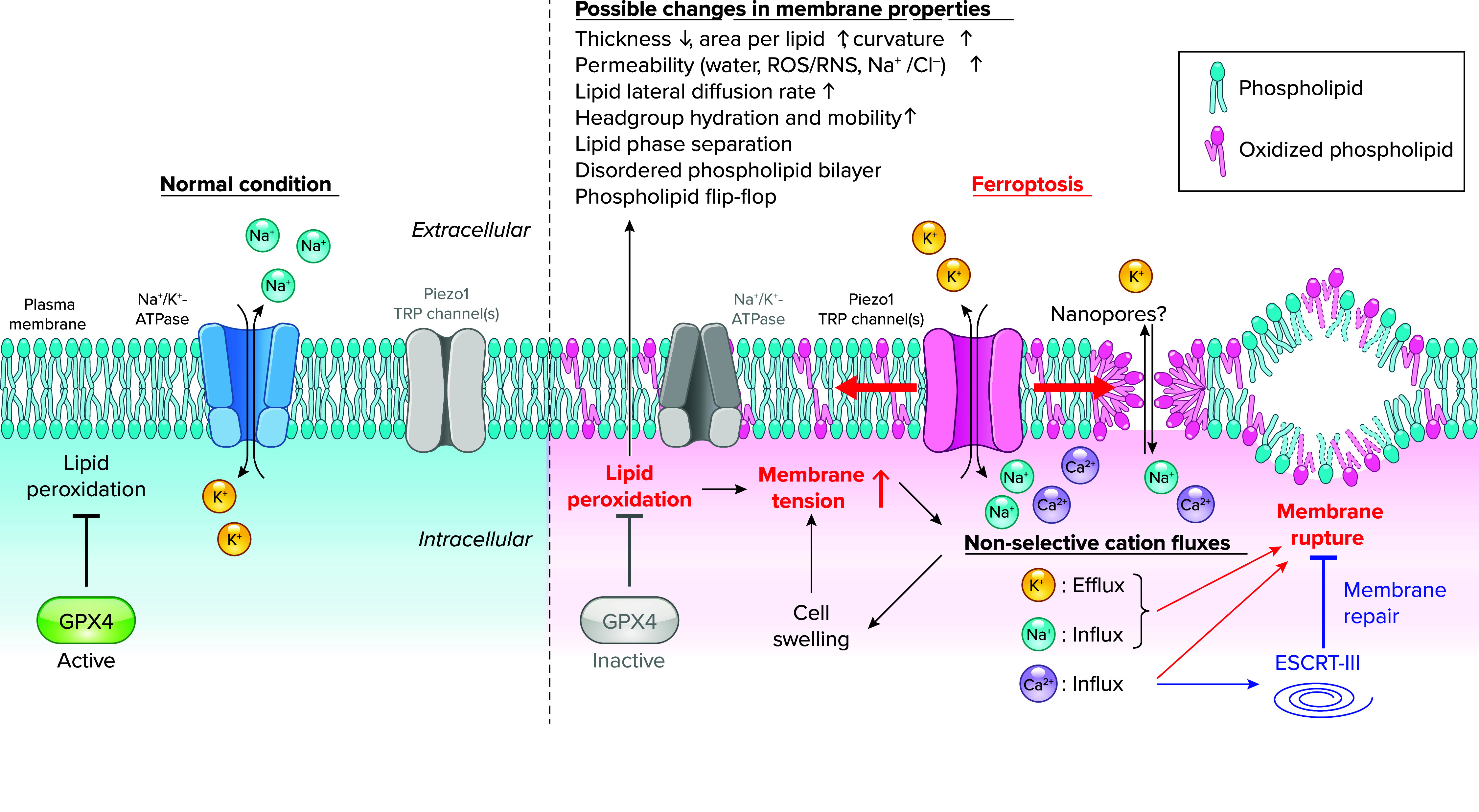
Membrane permeabilization and lipid peroxidation in ferroptosis Under normal conditions (*left*), glutathione peroxidase 4 (GPX4) plays a crucial role in suppressing lipid peroxidation for maintaining cell survival, and the concentration gradient of Na^+^/K^+^ across the plasma membrane is established mainly by Na^+^-K^+^-ATPase, a primary active transport system. Upon GPX4 inhibition (*right*), lipid peroxides accumulate throughout cells, particularly in the plasma membrane, potentially causing various changes in its membrane properties. Such changes lead to an increase in plasma membrane tension, thereby activating mechanosensitive cation channels, including Piezo1 and TRP channels while diminishing Na^+^-K^+^-ATPase activity. These collectively induce the collapse of cation gradients across the plasma membrane through drastic nonselective cation fluxes (namely K^+^ efflux, Na^+^ influx, and Ca^2+^ influx), resulting in cell swelling and further increase in plasma membrane tension. Nanopores might be formed in the plasma membrane during lipid peroxidation, contributing to cation fluxes as well. These cation fluxes may collectively promote membrane rupture. Ca^2+^ influx also plays a protective role in ferroptosis by triggering activation of the endosomal sorting complex required for transport (ESCRT-III), a membrane repair system. RNS, reactive nitrogen species; ROS, reactive oxygen species.

Lipid peroxidation, a process that can influence various aspects of lipid bilayers, is a defining characteristic of ferroptosis. Indeed, excessive phospholipid peroxidation for its cytotoxicity was not observed in other types of regulated cell death (35). Nevertheless, the precise mechanisms through which lipid peroxidation ultimately contributes to the execution of ferroptosis and cell membrane rupture remain to be elucidated, although molecular dynamics simulation studies indicated the effect of lipid peroxidation on membrane properties (FIGURE 2).

Molecular dynamics simulation has demonstrated that lipid peroxidation induces a diverse change in membrane properties, impacting the functions of membranes and membrane-associated proteins. For instance, molecular dynamics models using phosphatidylcholine (PC) and its primary oxidation products (peroxides) or secondary ones (alcohols and aldehydes) have revealed potential effects of lipid peroxidation on membrane properties (36–38). In an earlier study, the impact of lipid peroxidation on lipid bilayers was simulated with 1-palmitoyl-2-linoleoyl-*sn*-glycero-3-PC (16:0/18:2) (37). In detail, oxidation of the *sn*-2 linoleate chain, yielding hydroperoxides and aldehydes, led to a conformational change in the lipids. The oxidized tails bent toward the water phase, and the oxygen atoms formed hydrogen bonds with water and the polar lipid head group. These conformational changes resulted in an increase in the average area per lipid, leading to a decrease in bilayer thickness and an increase in water permeability. Lipid peroxidation was also suggested to induce faster lipid lateral diffusion and an increase in head group hydration and mobility (38). Experimental studies employing electron paramagnetic resonance spectrometry, nuclear magnetic resonance (NMR), and fluorescence spectroscopy have supported these simulations (38–40) and further demonstrated that lipid peroxidation can lead to lipid phase separation (41), disordered phospholipid bilayer (42), and phospholipid flip-flop (43). Recent molecular dynamics simulations have also demonstrated that peroxidation of membrane phospholipids increases their permeability to reactive oxygen species (ROS), reactive nitrogen species (RNS) (44, 45), and Na^+^/Cl^–^ ions (46). Notably, peroxidation of specific phospholipids, such as phosphatidylethanolamine (PE) with one arachidonyl (20:4) or one adrenyl (22:4) tail, has been reported to be more closely related to ferroptosis than other phospholipids (47). Molecular dynamics simulations specifically utilizing peroxidized PE containing linoleic acid (LA) or docosahexaenoic acid (DHA) have shown an increased area per lipid and curvature and a decreased thickness, in agreement with the previous study using PC (48). Taken together, lipid peroxidation induces diverse changes in membrane properties and affects various functions of membranes and membrane-associated proteins. However, it should be noted that these molecular dynamics simulations and experiments were based on extreme assumptions, and the composition of membrane lipids may not fully reflect their actual composition in the cellular context, especially considering that only a small fraction of phospholipid is oxidized by lipid peroxidation in the physiological setting (49). Consequently, it remains to be elucidated which of these changes in membrane properties are directly implicated in the execution of ferroptosis.

## Sensors and Effectors of Lipid Peroxidation in Membrane During Ferroptosis

Although several molecules have been reported as sensors and effectors responsible for detecting lipid peroxides and their derivatives, there remains limited knowledge regarding the identity of sensors responsible for detecting lipid peroxides in ferroptosis. Several receptors including scavenger receptors CD36 (50) and CD14 (51) and caspase-11 (52) have been reported for sensing oxidized 1-palmitoyl-2-arachidonoyl-*sn*-glycero-3-phosphocholines (oxPAPCs), which consist of a mixture of full-length and truncated oxidized PC derivatives. CD36 also binds diverse ligands beyond oxPAPCs, including oxidized low-density lipoprotein (oxLDL) and long-chain fatty acids, thereby promoting their uptake (53). In atherosclerosis, macrophage CD36 is known to play a role in promoting foam cell formation and inflammatory responses, exacerbating this disease (53). In a tumor microenvironment rich in PUFAs, CD36 facilitates uptake of PUFAs by tumor-infiltrating CD8^+^ T cells, which, in turn, promotes lipid peroxidation and ferroptosis. This leads to reduced cytotoxic cytokine production and antitumor effect (54). Thus, when cells are surrounded by proferroptotic lipids, such as oxidized lipids and PUFAs, CD36 can directly sense them and promote their incorporation, which in turn enhances ferroptosis.

CD14 is a receptor for bacterial lipopolysaccharide (LPS) on myeloid cells, and its interaction with oxPAPCs triggers their internalization into endosomes and transport to the cytosol, where they bind caspase-11 to induce a noncanonical inflammasome activation and subsequent secretion of IL-1β (51). Interestingly, myeloid-specific *Gpx4* depletion was shown to increase susceptibility to septic shock, due to lipid peroxidation-dependent activation of caspase-11 and cleavage of gasdermin D (GSDMD), which forms pores in the plasma membrane to execute pyroptosis, implicating a link of lipid peroxidation with inflammasome activation and pyroptosis (55).

Dead cells are normally detected and cleared by phagocytes such as macrophages, which recognize the characteristic “eat-me” signal exposed on their cell surface. A study demonstrated that macrophages engaged in the phagocytosis of ferroptotic dead cells by recognizing a specific oxidized phospholipid, namely 1-steaoryl-2–15-HpETE-*sn*-glycero-3-phosphatidylethanolamine, as an eat-me signal that becomes increased in the plasma membrane of ferroptotic cells (56). Additionally, the study proposed toll-like receptor 2 as a potential membrane receptor on macrophages responsible for detecting the oxidized lipid.

A recent report identified a unique role of the key caveola structural protein CAVIN1 as a sensor for membrane lipid peroxidation (57). CAVIN1 is predominantly localized at the plasma membrane as a component of caveolae. Upon oxidative stress conditions, such as exposure to hydrogen peroxide (H_2_O_2_) and GPX4 inhibition, it is released from the plasma membrane into the cytosol, which is inhibited by a lipophilic antioxidant, such as α-tocopherol. Once released to the cytosol, CAVIN1 binds to nuclear factor erythroid 2-related factor 2 (NRF2), a transcription factor for oxidative stress response. Consequently, the binding of CAVIN1 inhibits nuclear translocation of NRF2 and its transcriptional activity, thereby negatively regulating antioxidant responses. CAVIN1 deficiency conferred ferroptosis resistance in cancer cells and impaired a wound-healing process in zebrafish by inhibiting ROS generation and cell death (57). These results suggest that the caveola, an invaginated membrane structure in the plasma membrane with unique lipid composition and proteins including CAVIN1, might serve as a sensor and driver for lipid peroxidation, although the underlying mechanism of how it senses lipid peroxidation remains to be clarified.

## Lipid Peroxidation End Products

Lipid peroxidation generates end products, such as malondialdehyde (MDA) and 4-hydroxy-2-nonenal (4-HNE) (58, 59). Because of their stability compared to the primary and secondary lipid peroxidation products, they have been widely used as markers for lipid peroxidation (58, 59). MDA and 4-HNE are reactive aldehydes, forming adducts with various proteins and DNA, and are known to exert numerous deleterious effects on health (58, 59). Specific protein adducts with these aldehydes might be involved in ferroptosis execution, although direct evidence for this assumption is lacking. Detection of elevated levels of 4-HNE-modified protein, for example, by immunohistochemistry can be used as a surrogate indicator of lipid peroxidation. Nevertheless, relying solely on evidence of these end products, including 4-HNE, as indicators of ferroptosis is insufficient, as they lack specificity. They can also yield positive findings in nonferroptotic cells, such as those suffering oxidative stress.

## Roles of Cations in Regulated Cell Death

Cellular ion homeostasis is meticulously maintained by a diverse array of ion transporters and channel. Thus, the regulation and disruption of cellular ion level is involved in the process of each cell death modality. Among ions, cations are transported across the plasma membrane via ion pumps, known as primary active transporters, such as Na^+^-K^+^-ATPase and Ca^2+^-ATPase. This transport process establishes both electrical and chemical gradients, resulting in lower intracellular concentrations of Na^+^ and Ca^2+^ in comparison to their extracellular concentrations, whereas K^+^ maintains a much higher intracellular concentration (60, 61) (FIGURE 2). These gradients play a crucial role in mediating ionic signaling through ion channels and enable the transport of other solutes via secondary transporters. Dysregulation of ion homeostasis has been closely linked with various forms of regulated cell death, including apoptosis, necroptosis, pyroptosis, and ferroptosis (62, 63). In particular, accumulating evidence underscores the pivotal importance of the most abundant cations in the body, namely, Na^+^, K^+^, and Ca^2+^, in modulating the process of cell death. This section addresses the recent advances in the study of the roles of these cations in ferroptosis along with their importance in other forms of regulated cell death (FIGURE 2).

### Ferroptosis

#### Ca^2+^ channels.

Regarding the role of cations in ferroptosis, the involvement of Ca^2+^ has been extensively studied, but there are still unclear issues. In the earliest work, Ca^2+^ chelators, including BAPTA, Fura-2, and EGTA, failed to suppress erastin-induced ferroptosis in human sarcoma HT-1080 cells (6). In contrast, other studies demonstrated that BAPTA effectively blocks erastin-induced cell death in Lund human mesencephalic (LUHMES) neuronal-like cells (64) and that Ca^2+^ channel blockers, such as cobalt chloride and apomorphine, also prevented ferroptosis in the mouse hippocampal neuronal cell line HT-22 (65, 66). These contradictory findings may be attributed to variations in the experimental conditions and cell types, especially considering that LUHMES and HT-22 cells originate from neuronal cells. In addition, in evaluating the results of experiments involving inhibitor compounds, it is crucial to acknowledge that numerous compounds possessing radical scavenging potency inhibit ferroptosis in an off-target manner (30, 31). Consequently, when a channel inhibitor/activator prevents ferroptosis, it is imperative to meticulously assess whether the observed effect is a true on-target effect on the intended molecule or an off-target consequence attributable to radical scavenging ability.

The importance of Ca^2+^ has been also extensively explored in oxytosis, a type of oxidative stress-induced neuronal cell death that is now recognized as a part of the spectrum of ferroptosis (66). Oxytosis was originally characterized as a form of cell death induced by oxidative glutamate toxicity, distinct from excitotoxicity, which results from excessive extracellular glutamate stimulation of ionic glutamate receptors, thereby causing a massive influx of Ca^2+^ and neuronal death (8). However, excessive extracellular glutamate also inhibits cystine/glutamate transporter Xc^−^, leading to a decrease in intracellular GSH levels, similar to the events observed in ferroptosis induced by Xc^−^ inhibitors such as erastin (8). In addition, as glutamate-induced oxytosis is completely prevented by ferroptosis inhibitors, this type of cell death is now considered to be included in ferroptosis.

ROS is known to act as a potent inducer of store-operated Ca^2+^ entry (SOCE) through multiple mechanisms, for instance, endoplasmic reticulum (ER) Ca^2+^ store depletion through inositol 1,4,5-trisphosphate (IP_3_) receptor activation, sarco(endo)plasmic reticulum Ca^2+^-ATPase (SERCA) inhibition, and S-glutathionylation of stromal interaction molecule 1 (STIM1), which is the sensor protein for ER Ca^2+^ levels (8). Conversely, Ca^2+^ influx accelerates glutamate-induced ROS generation in mitochondria, primarily due to disrupted membrane potential and respiratory activities (67), ultimately promoting cell death. As a result, intracellular levels of Ca^2+^ and ROS increase in an interdependent manner, causing oxytosis (8). In HT-22 cells, ORAI calcium release-activated calcium modulator (Orai)1 and Orai3, members of the plasma membrane Ca^2+^ channels responsible for SOCE, likely contribute to Ca^2+^ influx during oxytosis, as their pharmacological or genetic inhibition protected against glutamate-induced cell death (66, 68). In addition, 4-HNE accumulated by lipid peroxidation may contribute to Ca^2+^ influx in hippocampal neurons by enhancing activation of voltage-dependent calcium channels (VDCCs) via their tyrosine phosphorylation (69). Overall, although it is generally accepted that Ca^2+^ influx through Ca^2+^ channels plays a causative role in oxytosis in neuronal cells, to date there is less evidence showing its involvement in general conditions in ferroptosis besides oxytosis. However, Orai1/3 knockdown partially suppressed RSL3-induced cell death in HT-22 cells, implying the contribution of these channels to ferroptosis (68).

Changes in extracellular temperature affect a series of cellular responses, including the state of the membrane and its channels. Indeed, cold stress induces ferroptosis. Cold stress-induced ferroptosis occurs in a manner dependent on a stress-responsive MAP kinase pathway, in particular the apoptosis signal-regulating kinase 1 (ASK1)-p38 pathway (70). This unique type of ferroptosis relies on mitochondrial calcium uptake 1 (MICU1) to facilitate Ca^2+^ uptake into mitochondria and trigger ROS generation (71). Transient receptor potential melastatin 8 (TRPM8) is also involved in Ca^2+^ influx across the plasma membrane in cold stress-induced ferroptosis (71). Of note, MICU1 deficiency or BAPTA, a Ca^2+^ chelator treatment, did not suppress erastin-induced ferroptosis, indicating that MICU1 is specifically required for cold stress-induced ferroptosis and that Ca^2+^ is not essential for ferroptosis induced by Xc^−^ inhibition (71). Thus, the role of Ca^2+^ in ferroptosis appears to be context dependent.

Interestingly, recent evidence has shown proferroptotic effects of Ca^2+^ through noncanonical mechanisms. A tetraspanin protein, membrane-spanning 4-domains subfamily A member 15 (MS4A15), drives lipid remodeling by depleting ER Ca^2+^ store, promoting accumulation of protective monounsaturated fatty acid-containing phospholipids and plasmalogen ether lipids while diminishing PUFA-containing phospholipids, thereby enhancing ferroptosis resistance (72). Intracellular Ca^2+^ levels are inversely correlated with the protein levels of Xc^−^ and GPX4 in vascular smooth muscle cells (73). Forced Ca^2+^ influx downregulates levels of Xc^−^ and GPX4, triggering ferroptosis, which in concert leads to vascular calcification associated with chronic kidney disease (73). Erianin, a natural compound isolated from *Dendrobium chrysotoxum Lindl,* exerts an antitumor activity by targeting calmodulin, a major Ca^2+^-binding protein involved in various Ca^2+^ signaling pathways (74). Erianin treatment elevated intracellular levels of Ca^2+^, ROS, and lipid peroxides while decreasing GSH levels, thereby inducing ferroptosis in cancer cells. Treatment with the calmodulin inhibitor calmidazolium reversed the proferroptotic effect of erianin, implicating a potential role of Ca^2+^/calmodulin signaling in the antitumor activity of erianin through its proferroptotic effect (74).

#### ESCRT-III.

Notably, a recent report implicated that phospholipid peroxidation creates nanoscale pores in the plasma membrane, leading to membrane permeability and allowing the influx of extracellular Ca^2+^ and water into cells. This ultimately results in cell swelling and membrane rupture, as these events were prevented by the presence of osmoprotectants such as polyethylene glycols (PEGs: PEG 6000–8000) (75). In addition, the study demonstrated that knockdown of charged multivesicular body protein 4B (CHMP4B), a component of the membrane repair system of endosomal sorting complex required for transport (ESCRT-III) complex, facilitated RSL3-induced ferroptosis, underscoring the significant role of ESCRT-III in suppressing ferroptosis (75). This finding is consistent with a report showing the importance of this membrane repair system in protecting cells against ferroptosis (76). However, another group reported different results, where treatment with PEGs (PEG 1450–3350) prevented cell swelling and membrane rupture but did not impede Ca^2+^ influx, which propagated throughout neighboring cells in a wavelike pattern, regardless of the presence of PEGs (75). Thus, further research is needed to determine whether nanoscale pores are actually formed in the plasma membrane and are responsible for Ca^2+^ influx during ferroptosis. Moreover, additional investigations are warranted to gain a comprehensive understanding of the potential roles of Ca^2+^ signaling and homeostasis in ferroptosis.

#### Piezo1 and TRP channels.

A recent report has unveiled key roles of mechanosensitive nonselective cation channels, including piezo-type mechanosensitive ion channel component 1 (Piezo1) (77) and TRP channels (78), in plasma membrane permeability of the monovalent cations, namely Na^+^ and K^+^, during ferroptosis (79) (FIGURE 2). Using BODIPY 581/591 C11 as an indicator for lipid peroxidation and a spinning-disk confocal microscope to minimize laser exposure, the study initially observed the preferential accumulation of lipid peroxides in the plasma membrane upon treatment with various ferroptosis inducers, determining plasma membrane as a key site of lipid peroxidation during ferroptosis. In response to RSL3, advanced three-dimensional (3-D) imaging techniques confirmed 10–20% increase in cell volume, which coincided with elevated plasma membrane tension, implicating a role of mechanosensitive channels, including Piezo1 and TRP channels. This study also found that a pan-cation channel inhibitor, ruthenium red, effectively inhibited ferroptosis induced by genetic Gpx4 depletion and pharmacological means (79). In accordance with this, genetic deletion of Piezo1 efficiently suppressed RSL3-induced ferroptosis without affecting lipid peroxidation. Atomic absorption spectrometry analysis revealed that GPX4 inhibition causes rapid Na^+^ influx and K^+^ efflux just before plasma membrane rupture, which was reversed by Piezo1 deficiency or elimination of lipid peroxides by ferrostatin-1 treatment. In parallel, Na^+^-K^+^-ATPase pump activity, which is critical for maintaining Na^+^/K^+^ concentration gradients across the plasma membrane, was also impaired after GPX inhibition. Importantly, blocking monovalent cation fluxes by substituting Na^+^ in the medium with *N*-methyl-d-glucamine^+^ (a cell-impermeant cation) or K^+^ prevented RSL3-induced ferroptosis, suggesting that Na^+^ influx and/or K^+^ efflux are involved in the execution of ferroptosis. In addition, a pan-TRP channel blocker, 2-aminoethyl diphenylborinate (2-APB), was shown to suppress ferroptotic cell death and cation fluxes, mimicking the effects of Piezo1 depletion. Moreover, Piezo1 depletion and 2-APB treatment synergistically inhibited ferroptosis, suggestive of a cooperative role of Piezo1 and TRP channels in cation fluxes and ferroptosis. These findings propose a model in which peroxidized lipids in the plasma membrane increase cell volume and membrane tension and trigger drastic Na^+^ influx/K^+^ efflux by activating Piezo1 and TRP channels, while impairing Na^+^-K^+^-ATPase activity. This, in turn, disrupts cation gradients across the plasma membrane, ultimately promoting plasma membrane rupture during ferroptosis (FIGURE 2).

This study offers several insights into a better understanding of membrane lipid peroxidation and ferroptosis. First, it directly demonstrates an increase in plasma membrane tension during ferroptosis, consistent with the previous molecular dynamics simulations (37, 48). Second, this research highlighted the occurrence of monovalent cation fluxes (Na^+^ influx and K^+^ efflux) before plasma membrane rupture, suggesting their contribution to ferroptosis. Third, in response to lipid peroxidation and subsequent elevation of plasma membrane tension, Piezo1 and TRP channels may act as mechanosensitive channels, facilitating cation permeability and thereby promoting ferroptosis. Of note, Piezo1 and TRP channels are known to function as Ca^2+^ channels, and the proferroptotic roles of Piezo1 as Ca^2+^ channels have also recently been reported by other groups (80, 81). Altogether, it is plausible that Piezo1 activation promotes ferroptosis through Na^+^/K^+^ fluxes and/or Ca^2+^ influx, depending on the context. Further studies are warranted to precisely determine the role of Piezo1 and TRP channels in ferroptosis.

#### Ninjurin1.

Ninjurin1 (NINJ1), a 16-kDa plasma membrane protein with two transmembrane domains, has been recently identified as a pore-forming protein inducing plasma membrane rupture at the final step of various types of cell death, including apoptosis-driven secondary necrosis, pyroptosis, and bacterial toxin-induced necrosis (82, 83). NINJ1 may also play a role in cation fluxes during these forms of cell death. However, the contribution of NINJ1 in plasma membrane rupture during ferroptosis has been controversial. One study reported that deletion of NINJ1 had no impact on RSL3-induced ferroptosis whereas it effectively suppressed cell rupture in apoptosis and pyroptosis (79), suggesting that the mechanism of plasma membrane rupture in ferroptosis may differ from other forms of regulated cell death. In contrast, another study reported that deletion of NINJ1 partially suppressed plasma membrane rupture in ferroptosis induced by GPX4 inhibitors, such as RSL3 and ML210 (84). However, this study also indicated that NINJ1 did not influence cell rupture in ferroptosis induced by other ferroptosis inducers, including erastin and FINO2, as well as genetic knockdown of GPX4. Therefore, the role of NINJ1 in ferroptosis appears to be context dependent, and further investigation is warranted.

### Apoptosis

**FIGURE 3.**
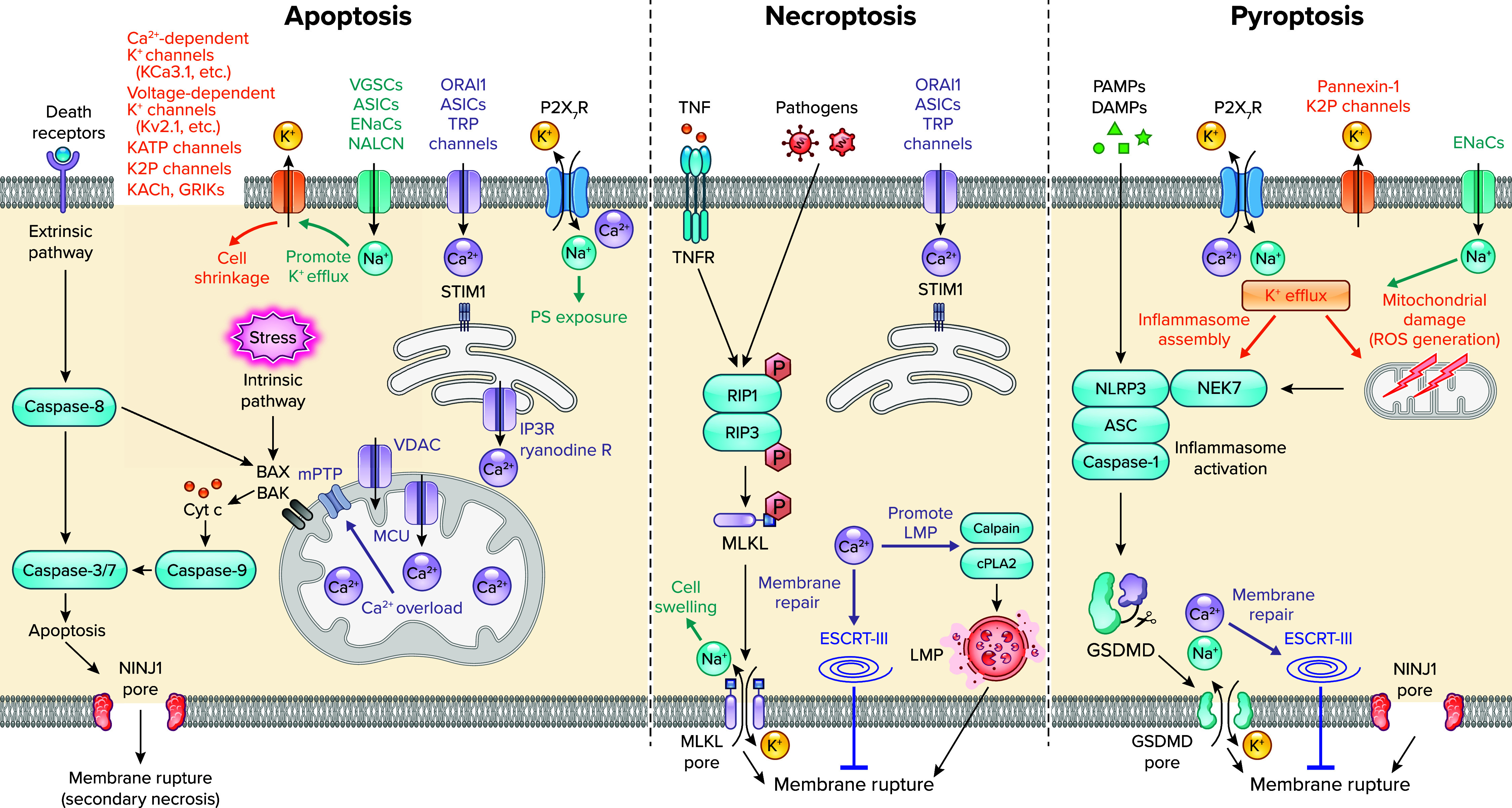
Cation handling during the process of each form of regulated cell death Regulated cell death encompasses diverse signaling pathways, each utilizing distinct and/or shared mechanisms in cation handling. *Left*: apoptosis is normally executed via 2 major signaling pathways: the extrinsic and intrinsic pathways. The extrinsic pathway is typically triggered by activation of death receptors, including tumor necrosis factor receptor (TNFR), leading to Caspase-8 activation, whereas the intrinsic pathway, initiated by numerous types of stresses including DNA damage, is induced by cytochrome *c* (Cyt c) release via B cell CLL/lymphoma-2 (BCL-2) family proteins, such as BCL-2-homologous antagonist/killer (BAK) and BCL-2-associated X protein (BAX), activating Caspase-9. Both pathways culminate in activation of Caspase-3/7, executing apoptosis. *Center*: necroptosis initiates with receptor-interacting protein (RIP)1/RIP3 activation in response to various stimuli, such as pathogens and TNFR activation, followed by mixed-lineage kinase domain-like protein (MLKL) phosphorylation by RIP3, leading to MLKL translocation to the plasma membrane and, ultimately, membrane rupture. *Right*: pyroptosis is triggered by danger-associated molecular patterns (DAMPs) and pathogen-associated molecular patterns (PAMPs,) activates inflammasomes such as NLRP3 inflammasome, consisting of NLRP3, apoptosis-associated speck-like protein containing a caspase recruitment domain (ASC), and Caspase-1, triggering cleavage of gasdermin D (GSDMD), resulting in plasma membrane pore formation and rupture. A wide variety of channels/pores participate in cation fluxes across membranes, occurring according to the concentration gradients, which play various roles in regulating each form of regulated cell death as indicated in the figure. See text for details. ASIC, acid-sensing ion channel; cPLA2, cytosolic phospholipase A_2_; ENaC, epithelial sodium channel; ESCRT-III, endosomal sorting complex required for transport; IP3, inositol 1,4,5-trisphosphate; LMP, lysosomal membrane permeabilization; MCU, mitochondrial calcium uniporter; mPTP, membrane permeability transition pore; PS, phosphatidylserine; R, receptor; ROS, reactive oxygen species; STIM1, stromal interaction molecule 1; VDAC, voltage-dependent anion channel; VGSC, voltage-gated sodium channel.

Ca^2+^ has been firmly established as a prominent proapoptotic factor, triggering so-called mitochondrial apoptosis. The accumulation of Ca^2+^ within mitochondria, referred to as Ca^2+^ overload, triggers the opening of mitochondrial membrane permeability transition pore (mPTP). This, in turn, increases the permeability of the mitochondrial membrane and facilitates the release of various apoptosis-inducing factors, including cytochrome *c* (85). Accordingly, cytochrome *c* binds to apoptotic protease activating factor 1 (APAF1), forming a multiprotein complex that recruits and activates the initiator caspase-9 through the caspase recruitment domain. The activation of caspase-9, in turn, cleaves and activates the executor caspases, including caspase-3, ultimately inducing apoptosis (85) (FIGURE 3).

A variety of channels participate in the process of Ca^2+^ overload. These include Ca^2+^ release channels such as IP_3_ and ryanodine receptors, located in the endoplasmic reticulum, as well as storage-type Ca^2+^ influx channels Orai1/Stim, acid-sensing ion channels (ASICs), and TRP channels, which are found in the plasma membrane (62, 86). Mitochondrial Ca^2+^ channels voltage-dependent anion channels (VDACs) and mitochondrial calcium uniporter (MCU) play major roles in transporting Ca^2 +^ across the outer and inner mitochondrial membranes, respectively (87). One characteristic feature of apoptosis is cell shrinkage, mainly resulting from the loss of cytosolic K^+^ and osmotically obliged water. This phenomenon is generally considered a driving force behind apoptosis (62). In addition, multiple types of K^+^ channels have been implicated in this process, such as Ca^2+^-dependent K^+^ channels, ATP-regulated K_ATP_ channels, voltage-gated K_v_ channels, and two-pore domain K^+^ (K2P) channels (62). In various studies, Na^+^ influx during apoptosis has been observed, and it has been shown to exert proapoptotic effects by promoting phosphatidylserine (PS) exposure through purinoceptor P_2X7_ activation in thymocytes (88) and by triggering K^+^ efflux through muscarinic K^+^ channel (KACh) (89) or G protein-gated inwardly rectifying K^+^ channel (GIRK, or Kir3) (90). Several other channels are known to be responsible for Na^+^ influx, such as voltage-gated sodium channels (VGSCs) and voltage-independent sodium channels like ASICs, epithelial sodium channels (ENaCs), and sodium leak channel NALCN (87) (FIGURE 3). However, the contribution of Na^+^ influx through these channels to apoptosis remains unclear and appears to be dependent on the cellular context.

### Necroptosis

Necroptosis is a form of regulated necrosis mediated by phosphorylation of receptor-interacting protein (RIP)1 and RIP3. This process is observed in various pathological conditions, such as pathogen infections (63). Activated RIP3 recruits and phosphorylates mixed-lineage kinase domain-like protein (MLKL), which translocates to the plasma membrane to form pores, eventually leading to lytic cell death (91, 92). During necroptosis, several cation channels, including store-operated Ca^2+^ influx channels (SOCE), ASICs, and TRP channels, open to induce Ca^2+^ influx (62, 93). MLKL translocation couples with activation of TRMP7, leading to Ca^2+^ influx and increased membrane rupture (94). Ca^2+^ influx can potentially activate calpain and cytosolic phospholipase A_2_, which results in lysosomal membrane permeabilization and subsequent leakage of lysosomal hydrolases, such as cathepsin family proteases, into the cytoplasm, leading to necrotic cell death (95). Simultaneously, it recruits the ESCRT-III complex to the plasma membrane to maintain its integrity, thereby counteracting necroptosis (96). Consequently, Ca^2+^ appears to play a multifaced role in necroptosis (FIGURE 3).

Importantly, MLKL has been shown to act not only as a pore but also as a cation channel with the following ion selectivity: *P*_Mg_ > *P*_Na_ ≈ *P*_K_ ≫ *P*_Ca_ and *P*_Cl_ (*P* represents permeability of the indicated cation) (97)_._ This likely accounts for the MLKL-dependent drastic Na^+^ influx observed before plasma membrane rupture, which increases intracellular osmolarity, causing cell swelling and bursting, representative markers of necroptosis (63). Although there is evidence suggesting the importance of K^+^ efflux in necroptosis induced by pore-forming toxins, further investigations are needed to fully understand its role (98).

### Pyroptosis

Pyroptosis is a caspase-dependent form of regulated cell death associated with inflammation. The term “pyroptosis” is derived from the Greek word *pyro*, meaning fire, signifying its inflammatory nature (99). In the canonical pathway, pyroptosis is initiated in response to various pathogen-associated molecular patterns and danger-associated molecular patterns (PAMPs and DAMPs) by activation of inflammasomes. These inflammasomes typically consist of three components: leucine-rich repeat-containing proteins (NOD-like receptors, NLRs) including NLR family leucine-rich repeat protein 1/3 (NLRP1/3) as a sensor protein, apoptosis-associated speck-like protein containing a caspase recruitment domain (ASC) as an adaptor protein, and pro-caspase-1 as an executor protein (99). An activated inflammasome induces the cleavage and activation of caspase-1, which, in turn, cleaves pro-IL-1β and pro-IL-18 for their maturation and also cleaves gasdermin D (GSDMD), a pore-forming protein. The NH_2_-terminal domain of GSDMD can oligomerize to form large pores in the plasma membrane, estimated to be 10–20 nm in diameter, resulting in plasma membrane rupture and release of mature IL-1β/IL-18 as proinflammatory cytokines (99) (FIGURE 3). Unlike MLKL in necroptosis, forming a selective cation channel (97) with smaller pores, ∼4 nm in diameter (100), GSDMD pore appears to have no ion selectivity because of the large pore size (10–20 nm in diameter) (101). It has been established that K^+^ efflux through multiple mechanisms, involving P_2X7_ receptor, pannexin-1, K2P channels, and GSDMD, is typically sufficient and necessary for NLRP3 inflammasome activation (102). K^+^ efflux is required for the interaction of NLRP3 with NIMA-related protein kinase 7 (NEK7), which is essential for the NLRP3 inflammasome assembly (103, 104). K^+^ efflux also leads to mitochondrial damage and subsequent ROS production, promoting NLRP3 inflammasome activation (105). As in the case of necroptosis, Ca^2+^ influx via GSDMD serves as a signal to recruit the membrane repair system ESCRT-III complex for negatively regulating IL-1β secretion and pyroptosis (106). Na^+^ influx through epithelial sodium channels (ENaCs) has been associated with enhanced K^+^ efflux and NLRP3 inflammasome activation, exacerbating cystic fibrosis (107).

## Ferroptosis and Disease

**FIGURE 4.**
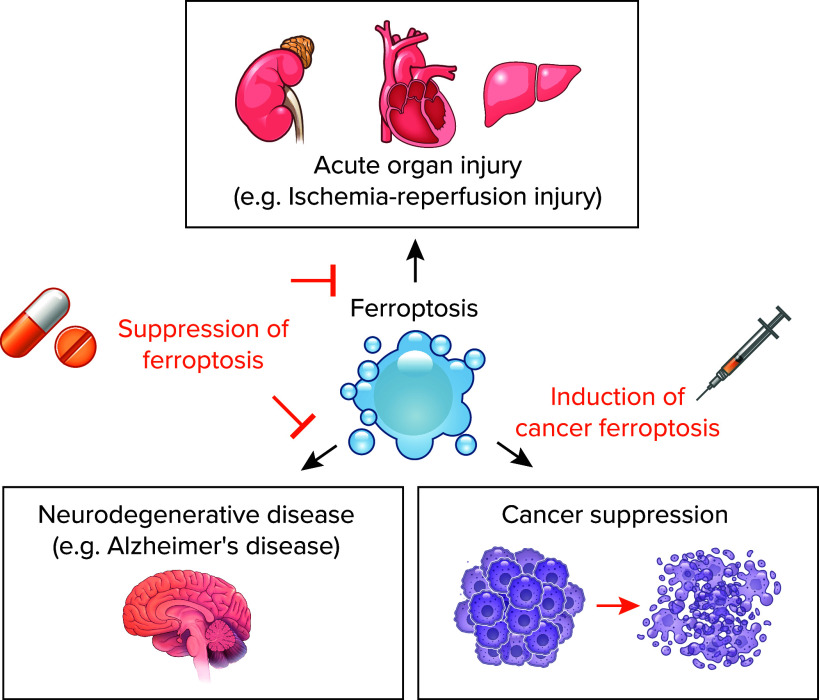
The role of ferroptosis in diverse disease contexts and the therapeutic approaches aimed at modulating this process Ferroptosis plays a contributory role in the pathophysiology of acute organ injury (e.g., ischemia-reperfusion), neurodegenerative diseases (e.g., Alzheimer’s disease), and cellular mechanisms involved in suppressing therapy-resistant cancers. Therapeutic strategies involve both the suppression of ferroptosis in organ injury and neurodegenerative diseases as well as the induction of ferroptosis in cancer cells.

Although the physiological role of ferroptosis remains elusive, extensive investigations have shed light on its significance in various pathologies, particularly in cancer suppression, acute organ injury, and neurodegenerative diseases (FIGURE 4) (5). Notably, the pharmacological modulation of ferroptosis has emerged as a promising therapeutic strategy for addressing these conditions in diverse preclinical animal models.

### Cancer

Ferroptosis is intricately linked to cancer, with numerous cancer-related genes and signaling pathways regulating ferroptosis (108). Compelling evidence suggests that targeting genes and pathways could provide new avenues for treating cancers (109). Intriguingly, therapy-resistant mesenchymal cancer cells and drug-tolerant persister cells have been shown to exhibit heightened dependence on the cyst(e)ine/GSH/GPX4 axis for their survival, rendering them susceptible to ferroptosis (12, 110). Thus, disrupting the endogenous defense systems that regulate ferroptosis provides an effective strategy for inducing ferroptosis in cancer cells. In the context of cancer pathophysiology, alteration in the function and expression of TRP channels and Piezo1 in cancer has been reported, impacting various cellular processes in tumors, such as differentiation, migration, and invasion (111–114). Thus, these changes may also affect the sensitivity of the cancer cells to ferroptosis. For instance, Piezo1 is activated upon mechanical stimulation and increases the intracellular Ca^2+^ level, leading to degradation of E-cadherin (115). Cells with loss of E-cadherin and subsequent inactivation of NF2 and hippo kinase have been reported to be susceptible to ferroptosis in cancer cells due to upregulation of multiple regulators of ferroptosis such as ACSL4 (116).

### Ischemia-Reperfusion Injury

Compelling evidence supports the notion that ferroptosis plays a pivotal role in cell death associated with ischemia-reperfusion injury (13, 117), induced by the cessation and subsequent restoration of blood flow to an organ. Ischemia followed by reperfusion triggers the generation of massive ROS, leading to lipid peroxidation (118). This, in turn, results in extensive cell death and inflammatory responses in affected organs, culminating in severe acute organ injuries, including brain stroke and ischemic heart disease as well as injuries to the liver and kidney. Ferroptosis inhibitors, mainly lipophilic radical trapping, have been successfully applied in animal models of ischemia-reperfusion-related tissue injuries including kidney, liver, heart, intestine, and brain (13, 19, 117, 119–121). Among the mechanisms contributing to the pathogenesis of ischemia-reperfusion injury, cytosolic Ca^2+^ is greatly exacerbated upon reperfusion (118). This Ca^2+^ overload would contribute to the execution of ferroptosis in the damage. Indeed, the potential role of TRPM channels and Piezo1 in ischemia-reperfusion injury has been reported (122, 123). For instance, *Trpm2*-knockout mice showed lower lipid peroxidation in renal ischemia-reperfusion damage (124). Increased expression of Piezo1 in a cerebral ischemic perfusion animal model has been reported (125).

### Neurodegenerative Diseases and Others

Ferroptosis has been implicated in neurodegenerative diseases, including Alzheimer’s disease, Parkinson’s disease, and amyotrophic lateral sclerosis (126), where a common feature is the progressive loss of neuronal cells. Lipid peroxidation and iron accumulation were found in these diseases long before ferroptosis was discovered and described (127). Genetic studies in mice have validated that conditional deletion of *Gpx4* can induce symptoms resembling neurodegeneration, such as cognitive impairment and motor neuron degeneration (128, 129). Although several clinical trials targeting ferroptosis through interventions like iron chelators and antioxidants have been conducted for neurodegenerative diseases, their beneficial effects have been controversial and have lacked convincing evidence thus far (130). Since the potential involvement of TRP channels and Piezo1 in the pathophysiology of neurodegenerative diseases has been reported (131, 132), these links may be associated with their effect on ferroptosis sensitivity. Furthermore, in other disease settings Piezo1, which facilitates calcium influx leading to increased sensitivity to ferroptosis, has been reported to play a role in regulating ferroptosis in the pathophysiology of radiation-induced lung injury and osteoarthritis (80, 81). In addition, Ca^2+^ channel TRPM7 regulated chondrocyte ferroptosis in a rheumatoid arthritis model (133).

### Ferroptosis and Inflammation

Ferroptosis is associated with inflammation signaling, which is also modulated by various ion channels, including Piezo1 and TRP channels (134, 135). Inflammatory signaling pathways, such as the nuclear factor-κB (NF-κB) pathway, the Janus kinase-signal transducer and activator of transcription (JAK-STAT) pathway, the cyclic GMP-AMP synthase-stimulator of interferon genes (cGAS)-stimulator of interferon response cGAMP interactor (STING) pathway, mitogen-activated protein kinase (MAPK) pathways, and the inflammasome pathway, either promote or suppress ferroptosis depending on the cell types and context (136). Thus, pharmacological intervention in these inflammatory signaling pathways has been reported to modulate ferroptosis sensitivity of the cells.

Deletion of leukemia inhibitory factor receptor (LIFR) conferred resistance to ferroptosis in hepatocytes and promoted liver tumorigenesis by enhancing activation of NF-κB signaling and thereby inducing lipocalin 2 transcription, decreasing cellular iron uptake (137). Dimethyl fumarate, an FDA-approved therapeutic agent for multiple sclerosis, alleviated neuroinflammation and ferroptosis possibly by decreasing activation of NF-κB signaling pathway, thereby improving cognitive impairment in a rat chronic cerebral hypoperfusion model (138). Interferon (IFN)-γ triggered activation of the JAK-STAT1-IRF1 pathway and suppressed system Xc^−^ expression in a transcriptional manner, which in turn inhibited xenograft tumor growth by promoting ferroptosis (139, 140). STAT3 has also been reported to promote ferroptosis in cancer cells (141, 142). The cytosolic DNA sensor cGAS is an activator for STING, playing a key role in inducing the type I IFN response (136). Intriguingly, STING has been reported as a driver of ferroptosis. Erastin triggers mitochondrial STING translocation in pancreatic cancer cells, where it binds to mitofusin 1/2 to accelerate mitochondrial fusion, leading to mitochondrial ROS production, lipid peroxidation, and ferroptosis (143). In a neonatal rat hypoxia-ischemia model, toll-like receptor 4 (TLR4)-p38 MAPK pathway was activated and induced the production of proinflammatory cytokines such as IL-1β, IL-6, and IL-18, while decreasing the expression of system Xc^−^ and GPX4, contributing to neuroinflammation and ferroptosis (144). Conversely, ferroptosis affects inflammatory signaling pathways, including the NLRP3 inflammasome pathway (136). Excessive iron potentiates NLRP3 inflammasome activation via the cGAS-STING pathway (142), whereas GPX4 blocks GSDMD cleavage and serves as a negative regulator (55). Octanol, a byproducts of lipid peroxidation, facilitates NLRP3 inflammasome activation (145), whereas an end product of lipid peroxidation, 4-HNE, binds to NLRP3 and prevents its interaction with NEK7, thereby suppressing its activation (146).

The immunogenicity of ferroptotic cells is a subject of debate. Necroptosis and pyroptosis, in which plasma membrane rupture occurs, are highly immunogenic and induce inflammation due to the release of proinflammatory cytokines and damage-associated molecular patterns (DAMPs), thereby promoting inflammatory response (63). Since ferroptosis also involves plasma membrane rupture, intracellular components, including DAMPs, are released into the extracellular space from the dead cells. Indeed, a study has reported that ferroptotic cells release DAMPs and inflammatory mediators (147). Nonetheless, the study also demonstrated less immunogenicity of ferroptotic dead cells compared to apoptosis and necroptosis. Mechanistically, the exertion of anti-inflammatory activity by specific types of oxidized phospholipids and prostaglandin E_2_, which increase during the process of ferroptosis (14), may contribute to the reduced immunogenicity of ferroptosis (148, 149). However, the precise physiological influence of ferroptosis in the complexity of the inflammatory response is yet to be fully elucidated.

## Perspectives

In the last decade, there has been dramatic progress in understanding the regulatory mechanisms and molecular aspects of ferroptosis. The development of therapeutics targeting ferroptosis shows great promise. Therefore, targeting cation channels and associated proteins involved in ferroptosis regulation is also warranted as a potential strategy for treating ferroptosis-associated diseases. However, key questions persist, such as *1*) the precise responsible signal that triggers ferroptosis, *2*) the mechanism by which lipid peroxidation leads to increased membrane permeability and plasma membrane rupture, and *3*) whether lipid peroxidation-induced radicals or another end product serve as the ultimate executor molecule; these aspects still require clarification.
